# Alcohol consumption and cancer incidence in women: interaction with smoking, body mass index and menopausal hormone therapy

**DOI:** 10.1186/s12885-023-11184-8

**Published:** 2023-08-16

**Authors:** Sarah Floud, Carol Hermon, Rachel F Simpson, Gillian K Reeves

**Affiliations:** 1 Cancer Epidemiology Unit, Nuffield Department of Population Health, University of Oxford, Oxford, UK; 2 MRC Epidemiology Unit, University of Cambridge, Cambridge, UK

**Keywords:** Alcohol, Cancer, Epidemiology

## Abstract

**Background:**

Alcohol consumption has been associated with increased risks of certain site-specific cancers and decreased risks of some other cancers. There is, however, little reliable evidence as to whether the alcohol-associated risks for specific cancers are modified by smoking, body mass index (BMI) and menopausal hormone therapy (MHT) use.

**Methods:**

In the prospective UK Million Women Study, 1,233,177 postmenopausal women without prior cancer, mean age 56 (SD 5) years, reported their alcohol consumption in median year 1998 (IQR 1998–1999), and were followed by record-linkage for incident cancer. 438,056 women who drank no alcohol or < 1 drink/week were excluded. Cox regression yielded adjusted relative risks (RRs) and 95% confidence intervals (CIs) for 21 cancers by alcohol amount; statistical significance of interactions with smoking, BMI and MHT use was assessed after allowing for multiple testing.

**Results:**

In 795,121 participants, mean consumption was 6.7 (SD 6.4) alcoholic drinks/week. During 17 (SD 5) years of follow-up, 140,203 incident cancers were recorded. There was strong evidence for a substantial association between alcohol intake and risk of upper aero-digestive cancers (oesophageal squamous cell carcinoma, oral cavity, pharynx and larynx; RR per 1 drink/day = 1.38 [95% CI 1.31–1.46]). There was also strong evidence for more moderate positive associations with breast, colorectal and pancreatic cancer (RRs per 1 drink/day = 1.12 [1.10–1.14], 1.10 [1.07–1.13], 1.08 [1.02–1.13] respectively), and moderate negative associations with thyroid cancer, non-Hodgkin’s lymphoma, renal cell carcinoma and multiple myeloma (RRs per 1 drink/day = 0.79 [0.70–0.89], 0.91 [0.86–0.95], 0.88 [0.83–0.94], 0.90 [0.84–0.97] respectively). Significant interactions between alcohol and smoking were seen for upper aero-digestive cancers (RRs per 1 drink/day = 1.66 [1.54–1.79], 1.23 [1.11–1.36], 1.12 [1.01–1.25] in current, past, and never smokers respectively). BMI and MHT did not significantly modify any alcohol-associated risks.

**Conclusions:**

These findings provide robust evidence that greater alcohol intake, even within relatively moderate ranges, increases the risk of cancers of the aerodigestive tract, breast, colorectal and pancreatic cancer, and probably decreases the risk of thyroid cancer, non-Hodgkin’s lymphoma, renal cell carcinoma and multiple myeloma. Associations of alcohol intake with cancer risk were not modified by MHT use, adiposity or smoking, except in the case of upper aero-digestive cancers, where the alcohol-associated risk was largely confined to smokers.

**Supplementary Information:**

The online version contains supplementary material available at 10.1186/s12885-023-11184-8.

## Background

The International Agency for Research on Cancer (IARC) and the World Cancer Research Fund (WCRF) have concluded there is ‘sufficient evidence’ from human and animal studies that consumption of alcohol causes cancers of the oral cavity, pharynx, oesophagus (squamous cell carcinoma), colon, rectum, liver and intrahepatic bile duct, larynx and female breast, and that there is a dose–response relationship between alcohol and these cancers, with no safe lower threshold [1]. There is, however, considerable uncertainty about other cancer sites, with only ‘probable evidence’ that alcohol consumption is associated with a higher risk of stomach cancer and a lower risk of kidney cancer, and ‘limited evidence’ that alcohol consumption is associated with cancers of the lung, pancreas and skin (basal cell carcinoma and malignant melanoma). Even in those cases where the association of alcohol with a specific cancer is well established, the mechanisms underlying such associations are still poorly understood [2]. For many cancers, a key mechanism is thought to be the conversion of ethanol by alcohol hydrogenase into carcinogenic acetaldehyde [1]. For other cancers, such as breast cancer, it has been hypothesised that greater alcohol intake increases risk by elevating levels of certain sex hormones [3–5].

It has also been suggested that the effect of alcohol intake on cancer risk may be modified by other behavioural risk factors for cancer, such as smoking, body mass index (BMI) and menopausal hormone therapy (MHT) use. However much of the previous evidence on interactions has been retrospective [6–8] and hence subject to recall bias (whereby there may be differential reporting of certain behaviours or exposures by individuals who have been diagnosed with cancer compared with those that have not). The existing evidence from prospective studies, where such biases are avoided or minimised, suggests that alcohol and smoking may have a synergistic effect on the risk of upper aerodigestive cancers, [9–11] although most studies have not found statistically significant interactions, possibly due to insufficient statistical power [12–16]. Findings from prospective studies on the alcohol-associated risks at other cancer sites have also been inconsistent regarding the potential modifying effect of either smoking, BMI or MHT use [3, 9, 10, 17–38]. An understanding of the impact of behavioural risk factors on alcohol-associated cancer risks is important for informing public health guidance, and may throw light on the biological mechanisms that underlie the effects of alcohol on specific cancers.

We have previously reported on the association between moderate alcohol intake and cancer incidence in the Million Women Study cohort, based on 68,775 cancers, diagnosed before 2007 and with an average follow-up of 7 years [9]. The present report is based on more than twice as many cases and an average follow-up per woman of 17 years, and includes a more detailed examination of alcohol-associated risks by smoking, BMI and MHT use.

## Methods

### Study population

The Million Women Study is a population-based prospective study [39]. In median year 1998 (IQR 1998–1999), women who were invited for National Health Service (NHS) breast cancer screening at 66 screening centres in England and Scotland, were invited to join the Million Women Study by completing a postal questionnaire about socio-demographic, anthropometric, health and lifestyle factors, including how much wine, beer, and spirits they drank on average each week. Subsequent surveys have been sent to participants every 3–5 years since recruitment. The study was approved by the East of England-Cambridge South Research Ethics Committee (97/5/001) and all participants provided written consent for follow-up through medical records. Study questionnaires and data access policies are available on the study website (www.millionwomenstudy.org).

### Ascertainment of incident cancer cases

All participants were followed by electronic record linkage to routinely collected NHS data on cancer registrations, deaths and emigrations. This enabled virtually complete follow-up (only 1.4% of the cohort have been lost to follow-up) [39]. Cancer diagnoses were coded to the 10^th^ revision of the World Health Organisation’s International Classification of Diseases. We examined the 21 most common cancers: oral cavity and pharynx (C00-C14), larynx (C32), stomach (C16), colorectum (C18-C20), liver (C22), pancreas (C25), lung (C34), malignant melanoma (C43), breast (C50), cervix (C53), endometrium (C54), ovary (C56), renal cell carcinoma (C64), bladder (C67), brain (C71), thyroid (C73), non-Hodgkin’s lymphoma (C82-C85), multiple myeloma (C90), and leukaemia (C91-C93, C95) and cancer of the oesophagus (C15), which was investigated separately by subtype, given the known differences in aetiology. Classification of oesophageal cancers by subtype was based on the following ICD-O morphology codes: oesophageal squamous cell carcinoma (OSCC): 8050–8086, and oesophageal adenocarcinoma (OAC): 8140–8145, 8190–8231, 8260–8263, 8310, 8315, 8480–8490, 8570–8575.

### Statistical analyses

The baseline for analyses of alcohol intake in relation to cancer risk was the recruitment questionnaire in median year 1998. Of the 1,364,268 women eligible for the analysis, 40,640 were excluded because they had a cancer (other than non-melanoma skin cancer, C44) registered before recruitment, 10,400 were excluded because they had missing information on alcohol consumption and, given the close correlation between alcohol consumption and smoking, a further 74,597 were excluded because their smoking status at baseline was not known. A further 438,056 women were excluded because they reported drinking no alcohol or less than 1 drink per week. This exclusion was done to account for the fact that most of the women who reported drinking less than one drink per week were ex-drinkers, [40] who may have stopped drinking due to poor health and could have different characteristics from drinkers, which might be difficult to measure but could be associated with the development of cancers [41]. Given that we were interested in the potential interaction of alcohol with MHT use, the analysis sample was further restricted to postmenopausal women (defined as those who reported that they had experienced natural menopause or had undergone bilateral oophorectomy). Women who were premenopausal, perimenopausal, or of unknown menopausal status at recruitment were assumed to be postmenopausal after they reached the age of 55 years and were included in follow-up from age 55 years, because 96% of women in this cohort with a known age at natural menopause were postmenopausal by that age. Therefore only 5454 women (who had not previously reported natural menopause or bilateral oophorectomy) were excluded either because they were diagnosed with cancer prior to 55 years old or because they exited the cohort before they reached 55 years old.

After exclusions, 795,121 women remained, for whom person-years were calculated from the date that the recruitment questionnaire was completed to the earliest of the following: first registration with cancer, death, emigration, or end of follow-up on 31^st^ December 2018. For analyses relating to cervical and endometrial cancers (*n* = 600,036), women were only included in the analysis if they had not reported a hysterectomy prior to recruitment. For analyses relating to ovarian cancer (*n* = 699,491), women were only included if they had not reported a bilateral oophorectomy prior to recruitment. For analyses relating to breast cancer (*n* = 756,462), a small proportion of women (4.9%) were excluded if they returned their recruitment questionnaire by post after their breast cancer screening appointment to ensure their answers were not influenced by any information gained at their screening appointment.

Women were grouped into four categories according to the total number of alcoholic drinks (i.e. 1 glass of wine, half a pint of beer/lager/cider, or 1 measure of spirits) reported at baseline: 1–2, 3–6, 7–14, and ≥ 15 drinks per week. Women who reported drinking 1–2 drinks per week were taken as the reference category. Cox proportional hazard models were used, with time since recruitment as the underlying time variable, to estimate the hazard ratios (henceforth referred to as relative risks [RRs]) and their 95% confidence intervals (CIs) for the various cancer sites. Analyses were stratified by year of birth and year of recruitment. Analyses were adjusted for the following variables recorded at recruitment: five regions of residence (London and Southeast, Southwest, Midlands, Northern England, Scotland), deprivation quintile (according to the Townsend index which is a score incorporating census area data for employment, car ownership, home ownership and household overcrowding [42]), educational qualifications (tertiary, secondary, technical, none), smoking (never, past, current < 5, 5–9, 10–14, 15–19, 20–24, ≥ 25 cigarettes per day), oral contraceptive use (never, ever), menopausal hormone therapy (MHT) use (never, past, current), BMI (< 20.0, 20.0–22.4, 22.5–24.9, 25–27.4, 27.5–29.9, 30.0–32.4, 32.5–34.9, ≥ 35 kg/m^2^), and strenuous physical activity (defined as exercise that is enough to cause sweating or a fast heart beat; never/rarely/ < once, once, 2–7 times per week). In order to include the same women in all analyses, a separate category was created for the small number of women with missing data for each adjustment variable (< 5% for each variable). For analyses relating to breast, ovarian, cervical and endometrial cancers, additional confounders were also included: age at menarche (< 12, 12–13, ≥ 14 years-old) and a combined variable of age at first birth (< 25, ≥ 25 years) and parity (nulliparous, 1-2, ≥ 3); and in breast cancer analyses, adjustment was also made for family history of breast cancer (yes, no). Potential violations of the proportional hazards assumption were assessed by examination of findings separately by 5 year follow-up periods. When comparing more than one group, group-specific 95% confidence intervals were calculated to allow direct comparison between any two groups [43].

The association of alcohol intake and cancer incidence was summarized in the form of a log-linear trend in risk per increase of one drink per day, and, for the purposes of this analysis, we considered a trend with *p* < 0.05 to be statistically significant. To correct for measurement error, including potential changes in intake over time, the test for trend across categories of intake was based on the mean alcohol intake (drinks/week) reported by 412,971 women at resurvey in median year 2006 (IQR 2006–2006) in each baseline alcohol intake category. We also conducted a sensitivity analysis excluding the first 5 years of follow-up, to assess whether any association was affected by reverse causality bias, whereby early symptoms of cancer might cause changes in alcohol intake.

The associations between alcohol and cancer incidence were further investigated in subgroups of women according to smoking status (never, past, current), BMI (< 25, 25–29, ≥ 30 kg/m^2^) and use of MHT (never, past, current) at baseline. Interactions between alcohol and the other factors were tested using a likelihood ratio test, and we adjusted for false discovery rate (FDR) using the Benjamini–Hochberg method, and an FDR threshold of 0.05 was set for significance. In analyses of interactions, all upper aerodigestive cancers (defined as cancers of the oral cavity, pharynx and larynx and OSCC) were combined and their interaction with smoking status at four levels (never, past, current < 10 cigarettes/day, current ≥ 10 cigarettes/day) was also calculated and presented graphically. For hormone-related cancers (breast, cervical, endometrial and ovarian), further analyses restricted the alcohol-BMI interaction to never users of MHT and restricted the alcohol-MHT interaction to women of normal BMI (20–24.9 kg/m^2^).

In order to assess the likely impact of reducing alcohol intake among women who reported drinking at least 1 drink per week, we estimated the proportion of aerodigestive, breast, colorectal, and pancreatic cancers, attributable to drinking more than 1–2 drinks per week, based on the distribution of women, and estimated relative risks by alcohol (and in the case of aerodigestive cancers by alcohol and smoking).

Stata version 17 (StataCorp, College Station, TX, USA) was used for all analyses. All statistical tests were two-sided.

## Results

A total of 795,121 women were included in this analysis, after the exclusions specified above. The participants were aged 56 years on average at recruitment and most reported a low to moderate level of alcohol consumption, with 92% drinking less than the current UK recommended limit of 14 drinks per week; 37% reported drinking 1–2 drinks per week, 26% reported drinking 3–6 drinks per week, 29% reported drinking 7–14 drinks per week, and 8% reported drinking greater than or equal to 15 drinks per week (Table 1). The mean alcohol intake was 6.7 drinks per week. Compared to those consuming lower levels of alcohol, women consuming higher levels of alcohol tended to have more educational qualifications, be physically active, have a lower BMI, be current smokers, be current users of MHT and have ever used the oral contraceptive pill.

**Table 1:** Characteristics of the study population at baseline, and details of follow-up, according to alcohol intake

	**Alcohol intake (drinks/week)**			
	**1–2**	**3–6**	**7–14**	** ≥ 15**
No. of women, n	292,249	209,275	230,749	62,848
Alcohol intake mean drinks/week (SD)^a^	2.6 (3.0)	5.5 (4.1)	10.0 (5.6)	17.0 (8.6)
Age, mean (SD)	56.7 (4.8)	56.3 (4.8)	56.1 (4.7)	55.8 (4.6)
Most deprived quintile, % (n)	17 (49,191)	17 (34,716)	16 (36,832)	16 (9,788)
No educational qualifications, % (n)	41 (117,865)	37 (76,584)	33 (74,749)	28 (17,625)
Current smoker, % (n)	16 (46,810)	18 (38,445)	22 (51,638)	26 (16,402)
Body mass index (kg/m^2^), mean (SD)	26.3 (4.6)	25.8 (4.2)	25.4 (4.0)	25.4 (4.1)
Current use menopausal hormones, % (n)	33 (96,504)	36 (74,486)	38 (87,214)	39 (24,432)
Ever use oral contraceptives, % (n)	59 (172,254)	64 (133,527)	69 (157,458)	73 (45,732)
Strenuous exercise > once per week, % (n)	20 (56,481)	23 (45,930)	25 (56,672)	28 (16,926)
**Follow-up**				
Person-years	4,992,142	3,552,867	3,873,456	1,030,747
Years of follow-up per woman	17.1	17.0	16.8	16.4
No. of cancers	50,555	36,139	41,325	12,184

^a^mean alcohol intake (drinks/week) in each category is the re-measured value taken on average 7.8 years after baseline

**Fig. 1:**
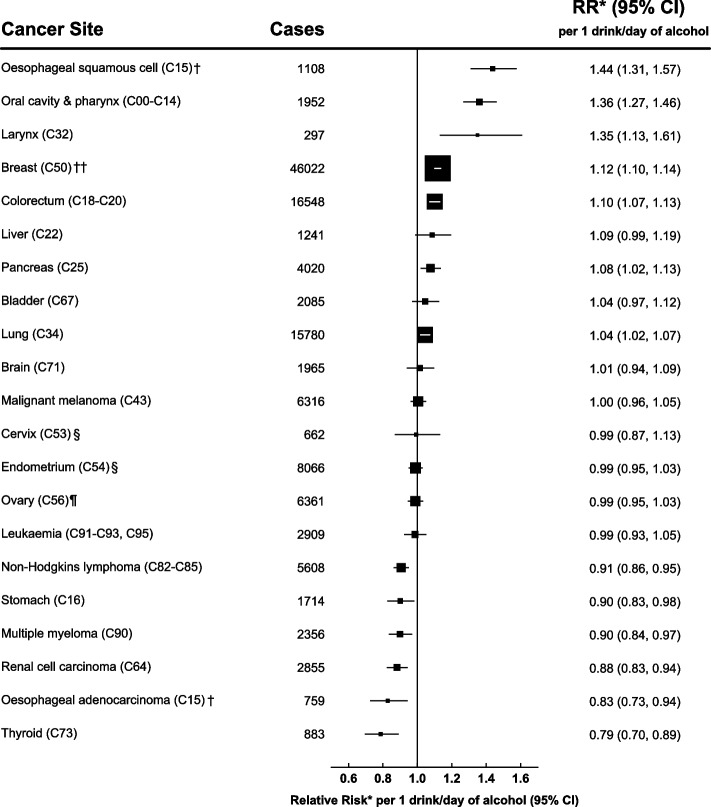
Relative risk by cancer site per 1 drink/day increase in alcohol intake * Relative risk and 95% confidence intervals (CI) per 1 drink/day increase in alcohol intake, adjusted for region, body mass index, deprivation index, educational attainment, smoking, strenuous exercise, use of oral contraceptives and menopausal hormones and stratified by year of birth and year completed recruitment questionnaire. For estimation of trends, intake categories 1-2, 3-6, 7-14, 15+ drinks/week were scored according to the mean alcohol intake at reassessment 7.8 years later (2.6, 5.5, 10.0, 17.0 drinks/week respectively). † See methods for histological classification. †† Additional adjustment for age at menarche, age at first birth, parity and family history of breast cancer. § Additional adjustment for age at menarche, age at first birth and parity. Includes only women who answered no to hysterectomy at recruitment. ¶ Additional adjustment for age at menarche, age at first birth and parity. Includes only women who answered no to bilateral oopherectomy at recruitment

Women were followed up for incident cancer over 13.4 million person-years, for an average of 16.9 years per woman (Table 1), over which time 140,203 incident cancers occurred. We calculated the relative risk of site-specific cancers by alcohol consumption at recruitment for every 1 drink per day increase in alcohol intake (Fig. 1) and by category of alcohol intake (eTable 1 in Additional file 1). An increasing amount of alcohol consumed was significantly associated with risks of OSCC (RR per 1 drink/day increase in alcohol intake = 1.44, 95% CI 1.31–1.57; *p* trend < 0.001), oral cavity and pharynx cancers (RR = 1.36, 1.27–1.46; *p* trend < 0.001), and larynx cancer (RR = 1.35, 1.13–1.61; *p* trend = 0.001). Given the similar elevated risks of the upper aerodigestive cancers (defined as cancers of the oral cavity, pharynx and larynx and OSCC) related to alcohol intake, we combined these cancers in subsequent analyses of interactions. The RR for these upper aerodigestive cancers per 1 drink per day increase in consumption was 1.38 (1.31–1.46); *p* trend < 0.001. There were also significant, but somewhat lesser, associations between alcohol and breast cancer (RR = 1.12, 1.10–1.14; *p* trend < 0.001), colorectal cancer (RR = 1.10, 1.07–1.13; *p* trend < 0.001), pancreatic cancer (RR = 1.08, 1.02–1.13, p trend = 0.006), and lung cancer (RR = 1.04, 1.02–1.07; p trend = 0.001). Alcohol intake was associated with an increased risk of liver cancer (RR = 1.09, 0.99–1.19) but this did not reach statistical significance (*p* trend = 0.083).

There were also decreased risks of certain cancers associated with alcohol intake. An increasing amount of alcohol consumed was most strongly associated with a decreased risk of thyroid cancer (RR = 0.79, 0.70–0.89; *p* trend < 0.001). There were lesser inverse associations for renal cell carcinoma (RR = 0.88, 0.83–0.94; *p* trend < 0.001), non-Hodgkin’s lymphoma (RR = 0.91, 0.86–0.95; *p* trend < 0.001), and multiple myeloma (RR = 0.90, 0.84–0.97; *p* trend = 0.004). There was also a decreased risk of OAC (RR = 0.83, 0.73–0.94; *p* trend = 0.004) but it should be noted that it was not possible in these analyses to adjust for gastric reflux, which is strongly associated with OAC risk and may also lead to a reduced intake of alcohol [44]. In fact, on a later questionnaire (median year 2006), participants were asked about gastric reflux symptoms and those who reported frequent symptoms of reflux or heartburn were more likely to report having reduced their alcohol intake due to illness (eTable 2 in Additional file 1). Therefore the apparent association between alcohol and OAC could be due to unmeasured confounding. The association between alcohol intake and stomach cancer (RR = 0.90, 0.83–0.98; *p* trend = 0.014) could also be affected by unmeasured confounding with acid reflux.

No significant increases or decreases related to alcohol intake were seen for the other cancers investigated, including bladder, leukaemia, cervical, malignant melanoma, endometrial, brain, and ovarian. There was no evidence of a material deviation from the proportional hazards assumption for any of the cancers. Sensitivity analyses were conducted for all cancers excluding the first 5 years of follow-up, but the alcohol-associated risks remained virtually the same (eTable 3 in Additional file 1).

**Fig. 2:**
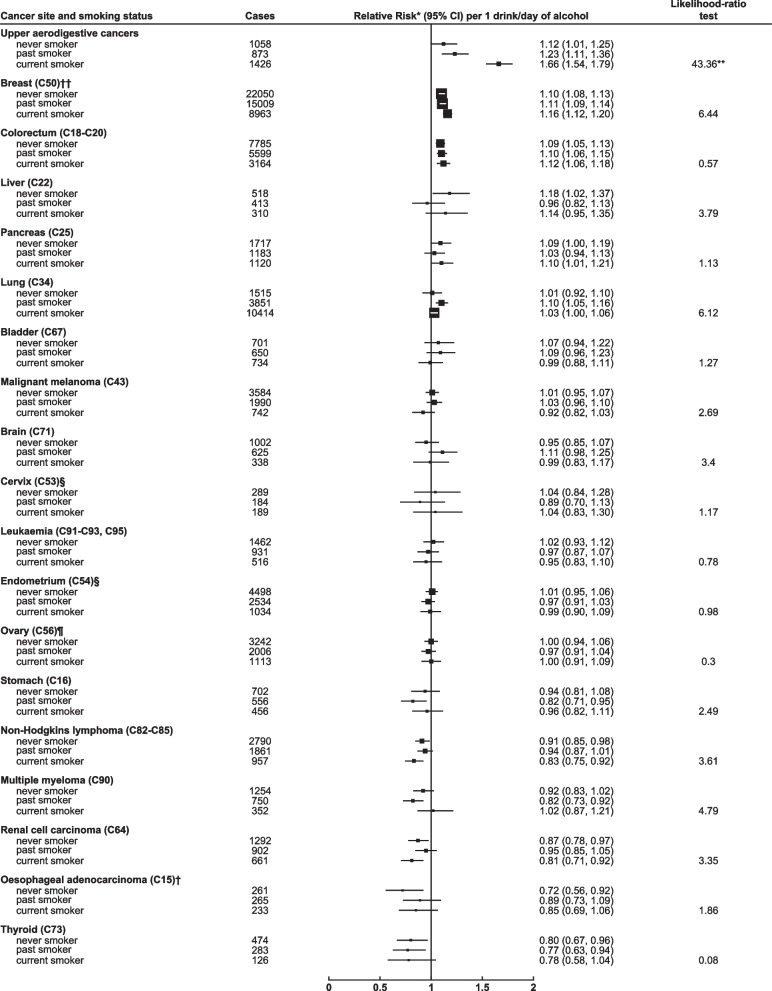
Relative risk by cancer site per 1 drink/day increase in alcohol intake by smoking status * Relative risk and 95% confidence intervals (CI) per 1 drink/day increase in alcohol intake, adjusted by region, body mass index, deprivation index, educational attainment, smoking (and number of cigarettes/day in current smokers), strenuous exercise, use of oral contraceptives and menopausal hormones and stratified by year of birth and year completed recruitment questionnaire. For estimation of trends, intake categories 1-2, 3-6, 7-14, 15+ drinks/week were scored according to the mean alcohol intake at reassessment 7.8 years later (2.6, 5.5, 10.0, 17.0 drinks/week respectively). ** denotes statistically significant at the FDR threshold (0.05). † See methods for histological classification. †† Additional adjustment for age at menarche, age at first birth, parity and family history of breast cancer. § Additional adjustment for age at menarche, age at first birth and parity. Includes only women who answered no to hysterectomy at recruitment. ¶ Additional adjustment for age at menarche, age at first birth and parity. Includes only women who answered no to bilateral oophorectomy at recruitment

**Fig. 3:**
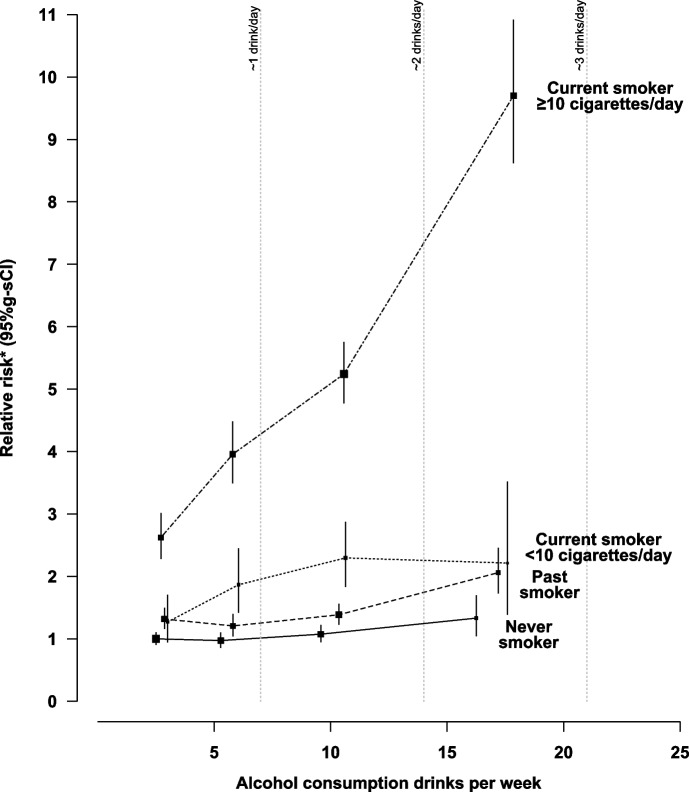
Relative risk of upper aerodigestive cancers by alcohol intake and smoking status * Relative risk and 95% g-s confidence intervals (CI) by amount of alcohol consumed and smoking compared to consumption of 1-2 drinks/week in never smokers (relative risk=1.0), adjusted for region, body mass index, deprivation index, educational attainment, strenuous exercise, use of oral contraceptives and menopausal hormones and stratified by year of birth and year completed recruitment questionnaire. The relative risks are for categories of one to two, three to six, seven-14, ≥ 15 drinks/week at recruitment plotted against the remeasured averages 7.8 years later in each of these categories (never smokers 2.5, 5.3, 9.6, 16.3 drinks/week respectively; past smokers 2.9, 5.8, 10.4, 17.2 drinks/week respectively; current smokers < 10 cigarettes/day 3.0, 6.0, 10.6, 17.6 drinks/week respectively; current smokers ≥ 10 cigarettes/day 2.7, 5.8, 10.6, 17.9 respectively). † Includes oesophageal squamous cell carcinoma, oral cavity and pharynx and larynx cancers

Figure 2 shows the adjusted RR for each cancer per 1 drink per day increase in consumption, in subgroups of women defined by their smoking status (never, past, current). The only interaction that was statistically significant at the corrected FDR threshold was that between smoking and alcohol on the risk of upper aerodigestive cancer: the RR per 1 drink/day increase in alcohol consumption was 1.66 (1.54–1.79) in current smokers, 1.23 (1.11–1.36) in past smokers and 1.12 (1.01–1.25) in never smokers. Smoking status was further split into four categories (never, past, current < 10 cigarettes/day, current ≥ 10 cigarettes/day) and the interaction between smoking and alcohol intake on the risk of upper aerodigestive cancer is shown in Fig. 3 (and eTable 4 in Additional file 1). Compared to the reference group of never smokers who only drank 1–2 drinks per week, the RR of upper aerodigestive cancer for never smokers who drank in excess of 14 drinks per week was 1.33 (1.05–1.69), the RR for past smokers who drank in excess of 14 drinks per week was more elevated at 2.06 (1.73–2.45), and the RR for current smokers who smoked ≥ 10 cigarettes/day and drank in excess of 14 drinks per week was substantially elevated at 9.70 (8.63–10.91).

There were no statistically significant interactions with smoking for any of the other cancer sites. For lung cancer, the significant alcohol-related RR per 1 drink/day of 1.04, 1.02–1.07, as shown in Fig. 1, was not evident in never smokers, with a RR per 1 drink/day increase in alcohol consumption of 1.01 (0.92–1.10) nor in current smokers (1.03, 1.00–1.06); instead the RR was most elevated among past smokers (1.10, 1.05–1.16), which may be due to lack of adjustment for number of cigarettes smoked in past smokers (as information on number of cigarettes smoked was not available for past smokers).

**Fig. 4:**
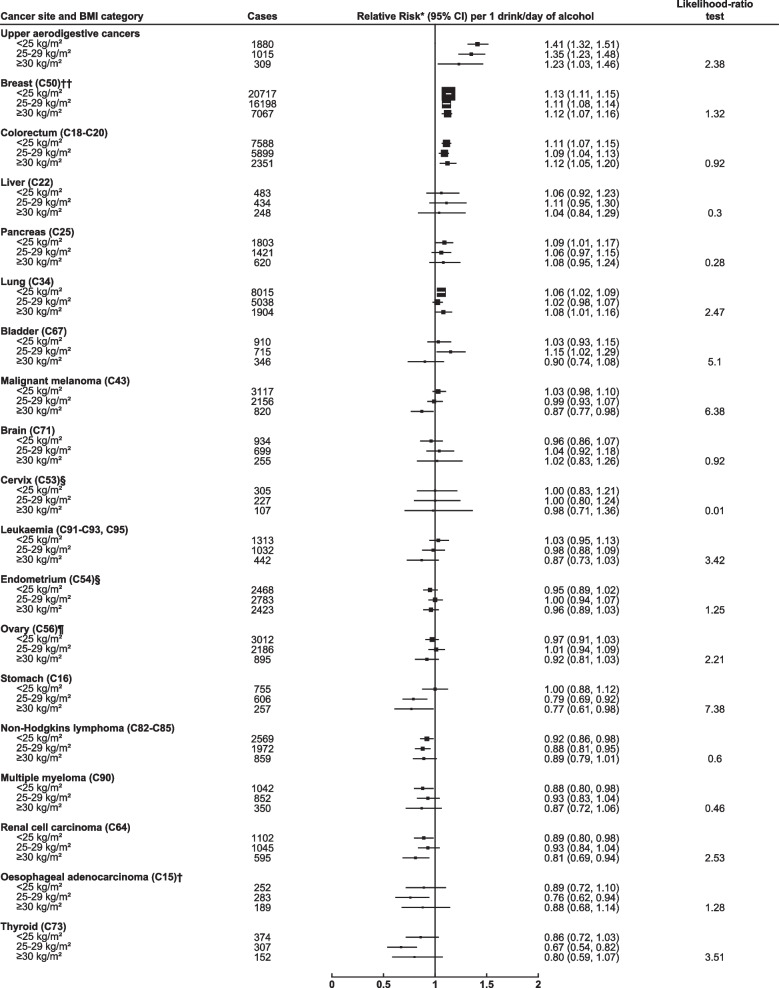
Relative risk by cancer site per 1 drink/day increase in alcohol intake by body mass index * Relative risk and 95% confidence intervals (CI) per 1 drink/day increase in alcohol intake, adjusted by region, deprivation index, educational attainment, smoking (and number of cigarettes/day in current smokers), strenuous exercise, use of oral contraceptives and menopausal hormones and stratified by year of birth and year completed recruitment questionnaire. For estimation of trends, intake categories 1-2, 3-6, 7-14, 15+ drinks/week were scored according to the mean alcohol intake at reassessment 7.8 years later (2.6, 5.5, 10.0, 17.0 drinks/week respectively). ** denotes statistically significant at the FDR threshold (0.05). † See methods for histological classification. †† Additional adjustment for age at menarche, age at first birth, parity and family history of breast cancer. § Additional adjustment for age at menarche, age at first birth and parity. Includes only women who answered no to hysterectomy at recruitment. ¶ Additional adjustment for age at menarche, age at first birth and parity. Includes only women who answered no to bilateral oophorectomy at recruitment

**Fig. 5:**
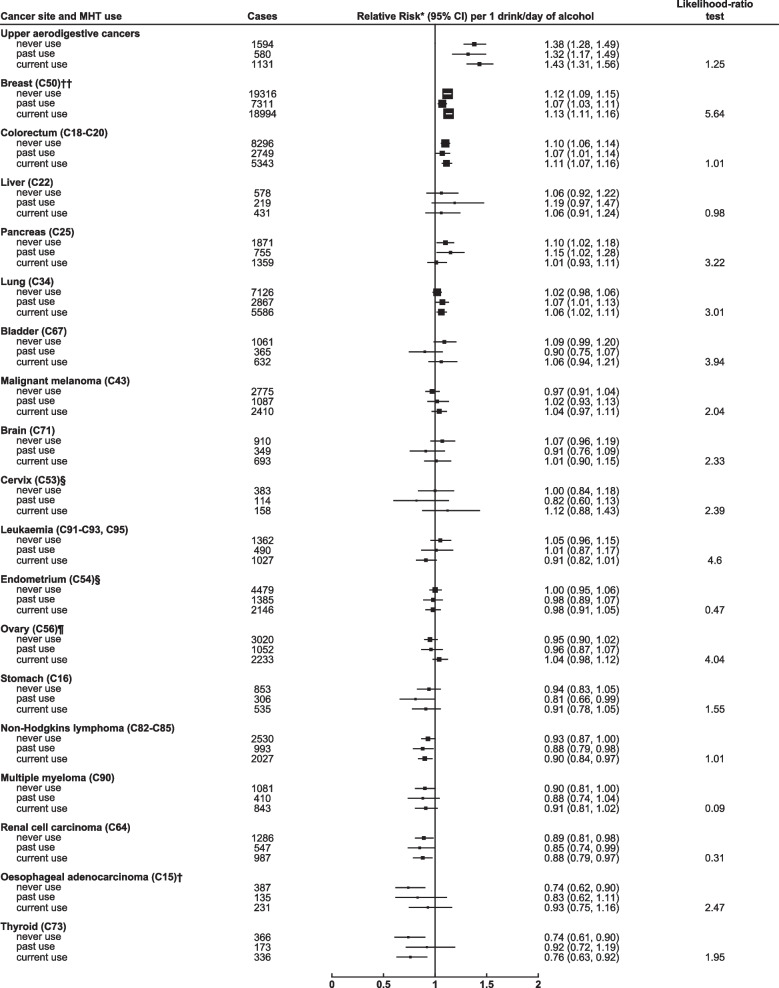
Relative risk by cancer site per 1 drink/day increase in alcohol intake by MHT use * Relative risk and 95% confidence intervals (CI) per 1 drink/day increase in alcohol intake, adjusted by region, body mass index, deprivation index, educational attainment, smoking (and number of cigarettes/day in current smokers), strenuous exercise and use of oral contraceptives and stratified by year of birth and year completed recruitment questionnaire. For estimation of trends, intake categories 1-2, 3-6, 7-14, 15+ drinks/week were scored according to the mean alcohol intake at reassessment 7.8 years later (2.6, 5.5, 10.0, 17.0 drinks/week respectively). ** denotes statistically significant at the FDR threshold (0.05). † See methods for histological classification. †† Additional adjustment for age at menarche, age at first birth, parity and family history of breast cancer. § Additional adjustment for age at menarche, age at first birth and parity. Includes only women who answered no to hysterectomy at recruitment. ¶ Additional adjustment for age at menarche, age at first birth and parity. Includes only women who answered no to bilateral oophorectomy at recruitment

Figures 4 and 5 show the RR for each cancer per 1 drink per day increase in alcohol consumption, in subgroups of women defined by their BMI (< 25, 25–29, ≥ 30 kg/m^2^) and use of MHT (never, past, current). After allowance for multiple testing, the alcohol-associated RR for each cancer did not vary significantly by BMI or by MHT use, nor was any heterogeneity detected for the hormone-related cancers when we restricted the alcohol-BMI analyses to never users of MHT and the alcohol-MHT analyses to women of normal BMI (20–24.9 kg/m^2^) (eTables 5 and 6 in Additional file 1).

Based on these findings, and taking into account the substantial interaction between smoking and alcohol for aerodigestive cancers, the proportion of site-specific cancers among women drinkers in this study population, that are attributable to drinking more than occasionally (defined here as an intake of 1–2 drinks per week), is estimated to be 21% (aerodigestive cancers), 6% (breast cancer), 5% (colorectal cancer), and 3% (pancreatic cancer). The absolute numbers of cases attributable to drinking more than occasionally will depend on the absolute incidence of each cancer.

## Discussion

In this large prospective cohort study of middle-aged women, most reported drinking much less than the current recommended limit of 14 drinks per week as was typical of women of this age [45]. We found convincing evidence that this moderate alcohol intake was associated with a substantial increased risk of cancers of the oral cavity, pharynx and larynx and oesophageal squamous cell carcinoma, and with a moderately increased risk of breast, colorectal, and pancreatic cancer. Moderate alcohol intake was also associated with a decrease in risk of thyroid cancer, renal cell carcinoma, non-Hodgkin’s lymphoma and multiple myeloma. While smoking greatly increased the alcohol-associated risks of cancers of the upper aerodigestive tract, neither BMI nor MHT use appeared to modify the association of alcohol with any of the cancers considered. Among middle-aged women drinkers, it is estimated that intakes of greater than 2 drinks per week account for around a fifth of aerodigestive cancers.

Compared to the conclusions drawn from the reviews by IARC and WCRF on alcohol-associated risks of cancer with ‘sufficient evidence’, [1], our findings concur for oral cavity, pharynx, larynx, OSCC, breast and colorectal cancer. However, we did not find a significant association with liver cancer, which is probably due to the relatively moderate intakes of alcohol which were typical of women in this cohort. For associations which had been classed as ‘probable’ in previous reviews, our findings add to the evidence for a slightly lower risk of renal cell carcinoma but do not provide support for an increased risk of stomach cancer related to alcohol, which again might be due to the relatively moderate intakes. It should be noted that for both liver cancer and stomach cancer, WCRF specified that the evidence was for intakes greater than about 3 drinks per day. For associations for which there was ‘limited evidence’, our findings provide some support for a slightly higher risk of pancreatic cancer, but do not provide any further support for a higher risk of malignant melanoma or lung cancer, as there was no association with malignant melanoma and the weak association observed with lung cancer was likely due to residual confounding by smoking, as there was no association with alcohol intake in never smokers. The reviews concluded that the evidence was inconsistent with regard to inverse associations between alcohol consumption and thyroid cancer, but together with similar results from a large Korean cohort [28], our findings add further large-scale evidence for an inverse association between alcohol and thyroid cancer [1]. Our findings also strengthen the existing evidence, which has been limited and inconsistent, for an inverse association between alcohol consumption and non-Hodgkin lymphoma and multiple myeloma, since the associations remained after excluding the first 5 years of follow-up.

### Interaction between alcohol and smoking

It has been suggested that tobacco and alcohol could have a synergistic effect on upper aerodigestive cancers because alcohol is thought to enhance the solubility, or the mucosal penetration, of tobacco carcinogens and thereby worsen the effect of smoking [46] but the prospective evidence has been uncertain. Interactions between alcohol and smoking with regard to specific upper aerodigestive sites, such as oesophageal squamous cell carcinoma, have been reported previously [10, 12–14] but most were not statistically significant when formally tested [12–14]. The prospective evidence is even more scarce for interactions between alcohol and smoking for cancers of the oral cavity, pharynx or larynx, with a statistically significant interaction between alcohol and smoking (p heterogeneity = 0.03) reported in the Netherlands Cohort Study for all oral cavity, pharynx and larynx cancers combined [11] and a non-significant interaction reported for oral cavity and pharynx cancer in the Japan Public Health Center cohort study [16]. Our findings clearly show that the majority of the alcohol-related excess risk of the upper aero-digestive cancers (oral cavity, pharynx, larynx and oesophageal squamous cell carcinoma) is in heavy smokers, with some excess risk in light smokers and past smokers and very little excess risk in never smokers, supporting the hypothesis that alcohol primarily acts by exacerbating the adverse effects of smoking [1]. In a previous analysis of the Million Women Study [9], the alcohol-related excess risk of upper aerodigestive cancer was only seen in current smokers, with little or no effect in never or past smokers. It is plausible that because of longer follow-up and the accrual of more cases in the analysis reported here, the small but significant alcohol-related excess risks for past smokers has emerged.

Interactions between alcohol and smoking in relation to other cancers have been investigated in several prospective studies, [17–25, 30] although significant interactions for only two cancer sites (thyroid and renal cell carcinoma) have been reported, to our knowledge [26–28]. However, the evidence for an interaction between alcohol and smoking on the risk of thyroid cancer is inconsistent, with one study reporting an alcohol-associated lower risk only in never smokers, [27] and the other, in a much larger study with 89,527 thyroid cases, reporting a lower risk in current smokers [28]. We found no evidence of an interaction between alcohol and smoking in relation to thyroid cancer, despite having a sufficiently large number of thyroid cases (*n* = 883). For all the other cancers, our findings, which are based on 100,000 more cancers than the largest previous study, did not suggest any substantial interaction between alcohol and smoking on cancer risk, other than for upper aerodigestive cancers, at least within the range of intakes observed in these middle-aged women.

### Interaction between alcohol and BMI

It has also been suggested that alcohol and obesity may act synergistically on the risk of certain cancers due to the principal alcohol metabolite, acetaldehyde, worsening the chronic inflammatory state related to obesity [8]. There have been reports from some case–control studies of higher alcohol-associated risks of colorectal and liver cancer, in heavier individuals [7, 8], but little evidence from prospective studies. This hypothesis is not supported by our findings nor by the evidence from other prospective studies on other cancers including oesophageal squamous cell carcinoma, renal cell carcinoma and malignant melanoma [10, 24, 26]. A pooled analysis of 14 cohort studies has reported a significant interaction for pancreatic cancer, with higher alcohol-associated risks in normal weight compared with overweight and obese participants, but it was only seen in men [47].

A further hypothesis has been proposed for the effect of alcohol and BMI on hormonal cancers. Since higher alcohol intake has been related to higher levels of circulating sex hormones [4, 5], the effect of alcohol on these cancers might be expected to be proportionately less in heavier women as they tend to already have higher circulating levels of sex hormones, since adipose tissue is the main site of oestrogen production in postmenopausal women [38]. There is some evidence from prospective studies of an effect of alcohol on risks of breast and endometrial cancer being confined to lean women, [35, 38] but other studies have not found such differences in risk by BMI [3, 19, 25, 29, 36]. In these data, there was no evidence of an elevated alcohol-associated risk of hormonal cancers in lean women, even after restricting our analyses to non-MHT users, which is in line with the findings from several smaller prospective studies [3, 19, 25, 29].

### Interaction between alcohol and MHT use

Like greater adiposity, MHT use is associated with higher levels of circulating oestrogens and so if alcohol acts on female cancers through increasing oestrogen, one might expect alcohol-associated risks to be proportionately greater in never users of MHT. However, several studies have suggested that MHT use may, in fact, increase the adverse effect of alcohol on the risk of these cancers [32, 33, 38]. We, along with other studies, [3, 20, 25, 29, 34, 37], did not find any evidence of interaction between alcohol and MHT on the risk of breast, endometrial, cervical or ovarian cancer, even after restricting our analyses to women of normal BMI.

### Strengths and limitations

The strengths of this study lie in its prospective design, the minimal loss to follow-up for cancers, and the large number of cases, all of which enabled us to thoroughly investigate interactions with other important lifestyle factors that could modify the associations between alcohol and cancer. In addition, the analysis was conducted only in alcohol drinkers, and those who reported drinking no, or only very little, alcohol were excluded. Using a reference group of women who drank 1–2 drinks per week means that the associations are less susceptible to bias, since those most likely to have quit drinking because of illness are not included in the reference group. Although we did not test formally for departures in log-linearity of associations of alcohol intake with cancer risk, the patterns of risk across categories of intake were broadly consistent with an assumption of log-linearity. A limitation of the study is that reporting of alcohol intake can be subject to measurement error, and women may change their intakes over time, although use of repeated measures of alcohol intake for estimation of dose response effects should have helped mitigate the latter type of bias. We were only able to study the effect of alcohol and its interactions in women and not in men. Men tend to drink more alcohol than women, which might lead to differences in attributable risk between men and women, but there is little evidence to suggest that trends in risk per drink differ materially by sex, as a comprehensive meta-analysis found no significant difference in risk by sex, except for one cancer [48]. It was not possible to investigate the effects of alcohol associated with all subtypes of the many cancers examined, but reports from this cohort have been published elsewhere investigating the association between alcohol consumption and subtypes of haematological malignancies [49].

## Conclusions

In this population of postmenopausal women, we have shown that alcohol intake is associated with an increased risk of certain cancers, even in a population where the vast majority are drinking less than the recommended weekly amount. Associations of alcohol intake with cancer risk were not modified by menopausal hormone use or adiposity or smoking, except in the case of upper aerodigestive cancers, the risks for which were substantially elevated for women who both drank alcohol and smoked.

## Supplementary Information

**Additional file 1:** **eTable 1.** Relative risks by cancer site for alcohol intake categories in drinks/week and per 1 drink/day of alcohol. **eTable2.** Frequency troubled by reflux/heartburn in relation to alcohol intake reported by 485,870 participants in median year 2006. **eTable 3.** Relative risks by cancer site for alcohol intake categories in drinks/week and per 1 drink/day of alcohol, excluding the first five years of follow-up. **eTable4.** RRs underlying Figure 3. **eTable5.** Alcohol-BMI interaction analyses restricted to never MHT users. **eTable6.** Alcohol-MHT interaction analyses restricted to women of normal BMI (20-24.9 kg/m²). 

## Data Availability

Anonymised data used here are available to any qualified researcher upon request to the investigators (addressed to mws.access@ndph.ox.ac.uk) and to the providers of follow-up data (eg, NHS England). The Million Women Study Data Access Policy can be viewed online at www.millionwomenstudy.org.

## Abbreviations

BMI: Body mass index
MHT: Menopausal hormone therapy
OSCC: Oesophageal squamous cell carcinoma
OAC: Oesophageal adenocarcinoma
