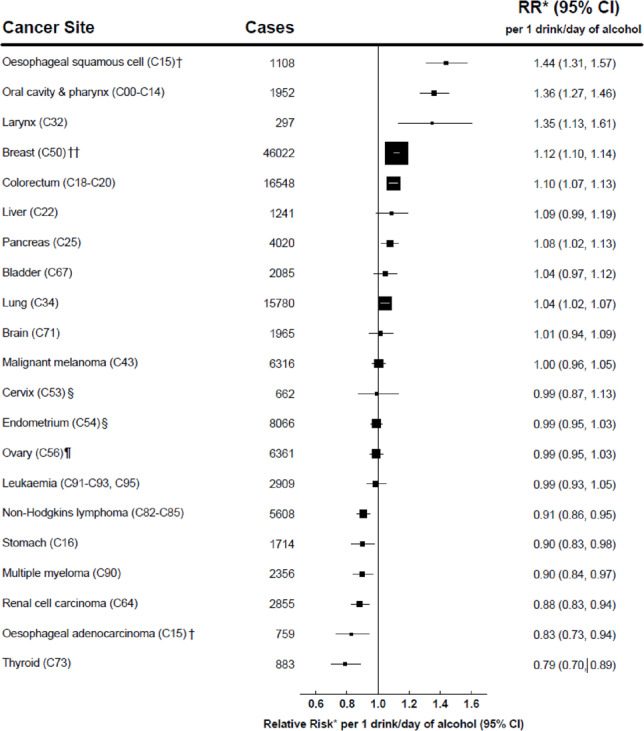# Correction to: Alcohol consumption and cancer incidence in women: interaction with smoking, body mass index and menopausal hormone therapy

**DOI:** 10.1186/s12885-026-15892-9

**Published:** 2026-03-31

**Authors:** Sarah Floud, Carol Hermon, Rachel F. Simpson, Gillian K. Reeves

**Affiliations:** 1 Cancer Epidemiology Unit, Nuffield Department of Population Health, University of Oxford, Oxford, UK; 2 MRC Epidemiology Unit, University of Cambridge, Cambridge, UK

**Correction: BMC Cancer 23**,** 758 (2023)**

**https://doi.org/10.1186/s12885-023-11184-8**

Following publication of the original article [1], the authors reported an error in Fig. 1. The number of breast cancer cases needs to be corrected to 46,022 (from 48540), and the upper confidence interval for the RR of breast cancer in relation to alcohol needs to be corrected to 1.14 (from 1.13).

Incorrect Fig. 1.


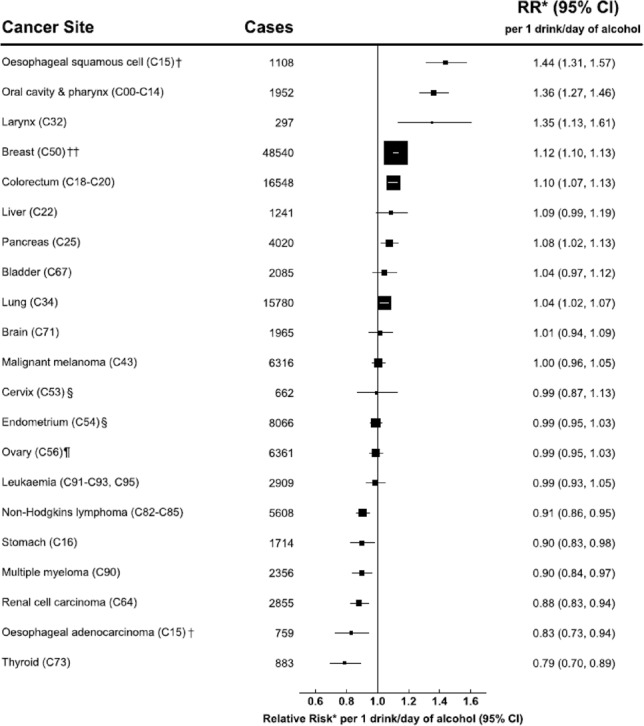


Correct Fig. 1.