# Supplementary material for: Alcohol consumption and cancer incidence in women: interaction with smoking, body mass index and menopausal hormone therapy
Source: BMC Cancer. 2023 Aug 16;23:758. doi: 10.1186/s12885-023-11184-8 (PMC10428611; doi:10.1186/s12885-023-11184-8)
Supplement: Supplementary file 1 — Additional file 1: eTable 1. Relative risks by cancer site for alcohol intake categories in drinks/week and per 1 drink/day of alcohol. eTable2. Frequency troubled by reflux/heartburn in relation to alcohol intake reported by 485,870 participants in median year 2006. eTable 3. Relative risks by cancer site for alcohol intake categories in drinks/week and per 1 drink/day of alcohol, excluding the first five years of follow-up. eTable4. RRs underlying Figure 3. eTable5. Alcohol-BMI interaction analyses restricted to never MHT users. eTable6. Alcohol-MHT interaction analyses restricted to women of normal BMI (20-24.9 kg/m²). [file 12885_2023_11184_MOESM1_ESM.pdf]

eTable 1: Relative risks by cancer site for alcohol intake categories in drinks/week and per 1 drink/day of alcohol

Cancer site	Alcohol consumption (drinks per week)																	RR* 95%CI per 1 drink/day of alcohol	p (trend)
		1-2				3-6				7-14				15+					
	N	n	RR*	95% g-sCI		n	RR*	95% g-sCI		n	RR*	95% g-sCI		n	RR*	95% g-sCI			
Oesophageal squamous cell carcinoma (C15)†	1108	317	1.00	0.89	1.12	255	1.10	0.97	1.25	372	1.38	1.24	1.53	164	2.13	1.82	2.50	1.44 (1.31-1.57)	<0.001
Oral cavity & pharynx (C00-C14)	1952	577	1.00	0.92	1.09	456	1.07	0.98	1.17	623	1.23	1.14	1.33	296	1.95	1.74	2.20	1.36 (1.27-1.46)	<0.001
Larynx (C32)	297	69	1.00	0.79	1.27	72	1.29	1.02	1.63	116	1.64	1.36	1.96	40	1.78	1.30	2.46	1.35 (1.13-1.61)	0.001
Breast (C50)††	46022	16013	1.00	0.98	1.02	11893	1.05	1.03	1.07	13920	1.12	1.11	1.14	4196	1.26	1.22	1.30	1.12 (1.10-1.14)	<0.001
Colorectum (C18-C20)	16548	5965	1.00	0.97	1.03	4270	1.03	1.00	1.06	4835	1.08	1.05	1.11	1478	1.25	1.18	1.31	1.10 (1.07-1.13)	<0.001
Liver (C22)	1241	457	1.00	0.91	1.10	321	1.03	0.92	1.15	356	1.06	0.96	1.18	107	1.21	1.00	1.47	1.09 (0.99-1.19)	0.083
Pancreas (C25)	4020	1466	1.00	0.95	1.05	1016	1.00	0.94	1.06	1197	1.08	1.02	1.15	341	1.15	1.03	1.28	1.08 (1.02-1.13)	0.006
Bladder (C67)	2085	735	1.00	0.93	1.08	558	1.08	0.99	1.17	621	1.08	0.99	1.17	171	1.09	0.93	1.26	1.04 (0.97-1.12)	0.233
Lung (C34)	15780	4982	1.00	0.97	1.03	3956	1.02	0.99	1.05	5183	1.05	1.02	1.08	1659	1.09	1.03	1.14	1.04 (1.02-1.07)	0.001
Brain (C71)	1965	722	1.00	0.93	1.08	533	1.05	0.96	1.14	559	1.02	0.94	1.11	151	1.04	0.89	1.23	1.01 (0.94-1.09)	0.701
Malignant melanoma (C43)	6316	2280	1.00	0.96	1.04	1759	1.08	1.03	1.13	1810	1.03	0.98	1.08	467	1.01	0.92	1.11	1.00 (0.96-1.05)	0.838
Cervix (C53)§	662	241	1.00	0.88	1.14	182	1.07	0.92	1.23	184	0.97	0.84	1.12	55	1.04	0.79	1.36	0.99 (0.87-1.13)	0.907
Endometrium (C54)§	8066	3327	1.00	0.97	1.04	2075	0.96	0.92	1.00	2071	0.94	0.90	0.98	593	1.03	0.95	1.11	0.99 (0.95-1.03)	0.562
Ovary (C56)¶	6361	2473	1.00	0.96	1.04	1612	0.93	0.89	0.98	1779	0.95	0.91	1.00	497	1.00	0.91	1.09	0.99 (0.95-1.03)	0.587
Leukaemia (C91-C93, C95)	2909	1116	1.00	0.94	1.06	762	0.99	0.92	1.06	825	1.00	0.93	1.07	206	0.95	0.83	1.09	0.99 (0.93-1.05)	0.663
Non-Hodgkins lymphoma (C82-C85)	5608	2250	1.00	0.96	1.04	1482	0.95	0.90	1.00	1512	0.90	0.85	0.94	364	0.82	0.74	0.91	0.91 (0.86-0.95)	<0.001
Stomach (C16)	1714	693	1.00	0.93	1.08	449	0.93	0.85	1.02	463	0.89	0.81	0.97	109	0.81	0.67	0.98	0.90 (0.83-0.98)	0.014
Multiple myeloma (C90)	2356	986	1.00	0.94	1.07	596	0.88	0.81	0.95	627	0.88	0.81	0.95	147	0.80	0.68	0.94	0.90 (0.84-0.97)	0.004
Renal cell carcinoma (C64)	2855	1163	1.00	0.94	1.06	759	0.94	0.88	1.01	754	0.87	0.81	0.94	179	0.78	0.67	0.90	0.88 (0.83-0.94)	<0.001
Oesophageal adenocarcinoma (C15)†	759	335	1.00	0.90	1.12	189	0.82	0.71	0.94	180	0.71	0.61	0.82	55	0.80	0.61	1.05	0.83 (0.73-0.94)	0.004
Thyroid (C73)	883	383	1.00	0.90	1.11	233	0.86	0.76	0.98	216	0.74	0.65	0.85	51	0.65	0.49	0.86	0.79 (0.70-0.89)	<0.001

*Adjusted for region, body mass index, deprivation index, educational attainment, smoking, strenuous exercise, use of oral contraceptives and menopausal hormones and stratified by year of birth and year completed recruitment questionnaire. For estimation of trends, intake categories 1-2, 3-6, 7-14, 15+ drinks/week, were scored according to the mean alcohol intake at reassessment 7.8 years later in each baseline group (2.6, 5.5, 10.0, 17.0 drinks/week respectively).

† See methods for histological classification.

†† Additional adjustment for age at menarche, age at first birth, parity and family history of breast cancer.

§ Additional adjustment for age at menarche, age at first birth and parity. Includes only women who answered no to hysterectomy at recruitment.

¶ Additional adjustment for age at menarche, age at first birth and parity. Includes only women who answered no to bilateral oophorectomy at recruitment.

eTable2: Frequency troubled by reflux/heartburn in relation to alcohol intake reported by 485,870 participants in median year 2006

	Frequency troubled by reflux/heartburn			
	rarely/never	< once week	about once week	> once week
Drinks/week, mean (SD) (n)	5.4 (6.3) (299702)	5.2 (6.1) (79215)	5.0 (6.1) (42432)	4.5 (6.0) (64521)
Reduced alcohol intake due to illness, % (n)	4.7 (13926)	6.0 (4715)	7.9 (3344)	10.3 (6638)

eTable 3: Relative risks by cancer site for alcohol intake categories in drinks/week and per 1 drink/day of alcohol, excluding the first five years of follow-up

Cancer site	Alcohol consumption (drinks per week)																		RR* 95%CI per 1 drink/day of alcohol	p [(trend)]
	1-2					3-6				7-14				15+						
	N	n	RR*	95% g-sCI		n	RR*	95% g-sCI		n	RR*	95% g-sCI		n	RR*	95% g-sCI				
Oesophageal squamous cell carcinoma (C15)†	915	266	1.00	0.88	1.13	213	1.10	0.96	1.26	306	1.36	1.21	1.52	130	2.04	1.71	2.44		1.41 (1.27-1.56)	<0.001
Oral cavity & pharynx (C00-C14)	1595	463	1.00	0.91	1.10	370	1.08	0.98	1.20	518	1.29	1.18	1.41	244	2.04	1.80	2.33		1.40 (1.30-1.51)	<0.001
Larynx (C32)	230	57	1.00	0.77	1.30	55	1.19	0.91	1.55	86	1.47	1.19	1.81	32	1.76	1.23	2.52		1.33 (1.09-1.62)	0.005
Breast (C50)††	34053	11895	1.00	0.98	1.02	8803	1.05	1.03	1.07	10272	1.13	1.11	1.15	3083	1.27	1.23	1.32		1.12 (1.10-1.14)	<0.001
Colorectum (C18-C20)	13891	4976	1.00	0.97	1.03	3595	1.04	1.01	1.08	4085	1.10	1.06	1.13	1235	1.26	1.19	1.33		1.11 (1.08-1.14)	<0.001
Liver (C22)	1121	414	1.00	0.91	1.10	294	1.05	0.93	1.17	319	1.06	0.95	1.19	94	1.19	0.97	1.46		1.08 (0.97-1.19)	0.148
Pancreas (C25)	3521	1282	1.00	0.95	1.06	901	1.02	0.95	1.09	1037	1.08	1.01	1.15	301	1.17	1.04	1.31		1.08 (1.02-1.14)	0.007
Bladder (C67)	1745	612	1.00	0.92	1.08	463	1.07	0.98	1.17	527	1.09	1.00	1.19	143	1.09	0.92	1.28		1.05 (0.97-1.14)	0.216
Lung (C34)	13698	4330	1.00	0.97	1.03	3452	1.02	0.99	1.06	4481	1.05	1.02	1.08	1435	1.09	1.04	1.15		1.04 (1.02-1.07)	0.002
Brain (C71)	1582	590	1.00	0.92	1.09	417	1.00	0.91	1.10	455	1.01	0.92	1.10	120	1.01	0.84	1.21		1.00 (0.92-1.09)	0.915
Malignant melanoma (C43)	5126	1837	1.00	0.95	1.05	1437	1.10	1.04	1.16	1489	1.06	1.00	1.11	363	0.99	0.89	1.10		1.01 (0.96-1.06)	0.771
Cervix (C53)§	465	170	1.00	0.86	1.17	127	1.06	0.90	1.27	132	1.00	0.84	1.19	36	1.01	0.72	1.40		1.00 (0.85-1.16)	0.950
Endometrium (C54)§	6432	2656	1.00	0.96	1.04	1651	0.96	0.91	1.00	1658	0.95	0.90	0.99	467	1.02	0.93	1.11		0.99 (0.95-1.03)	0.580
Ovary (C56)¶	4775	1851	1.00	0.95	1.05	1233	0.95	0.90	1.01	1319	0.94	0.89	1.00	372	1.01	0.91	1.12		0.99 (0.94-1.03)	0.558
Leukaemia (C91-C93, C95)	2468	958	1.00	0.94	1.07	640	0.97	0.89	1.04	701	0.99	0.92	1.07	169	0.92	0.79	1.08		0.98 (0.91-1.05)	0.513
Non-Hodgkins lymphoma (C82-C85)	4666	1843	1.00	0.95	1.05	1234	0.96	0.91	1.02	1289	0.94	0.89	0.99	300	0.83	0.74	0.93		0.93 (0.88-0.97)	0.003
Stomach (C16)	1394	564	1.00	0.92	1.09	371	0.95	0.86	1.05	375	0.90	0.81	0.99	84	0.79	0.63	0.98		0.89 (0.81-0.98)	0.020
Multiple myeloma (C90)	2030	866	1.00	0.93	1.07	511	0.86	0.79	0.94	527	0.85	0.78	0.92	126	0.79	0.66	0.94		0.88 (0.81-0.95)	0.001
Renal cell carcinoma (C64)	2412	991	1.00	0.94	1.07	651	0.95	0.88	1.03	617	0.84	0.77	0.91	153	0.79	0.67	0.92		0.87 (0.81-0.93)	<0.001
Oesophageal adenocarcinoma (C15)†	678	302	1.00	0.89	1.12	162	0.78	0.67	0.91	164	0.72	0.62	0.84	50	0.82	0.62	1.08		0.84 (0.74-0.97)	0.013
Thyroid (C73)	714	308	1.00	0.89	1.12	186	0.86	0.74	0.99	178	0.76	0.65	0.88	42	0.67	0.49	0.90		0.80 (0.70-0.92)	0.001

*Adjusted for region, body mass index, deprivation index, educational attainment, smoking, strenuous exercise, use of oral contraceptives and menopausal hormones and stratified by year of birth and year completed recruitment questionnaire. For estimation of trends, intake categories 1-2, 3-6, 7-14, 15+ drinks/week, were scored according to the mean alcohol intake at reassessment 7.8 years later in each baseline group (2.6, 5.5, 10.0, 17.0 drinks/week respectively).

† See methods for histological classification.

†† Additional adjustment for age at menarche, age at first birth, parity and family history of breast cancer.

§ Additional adjustment for age at menarche, age at first birth and parity. Includes only women who answered no to hysterectomy at recruitment.

¶ Additional adjustment for age at menarche, age at first birth and parity. Includes only women who answered no to bilateral oophorectomy at recruitment.

eTable4: RRs underlying Figure 3

Alcohol consumption (drinks per week)	Smoking at recruitment															
	Never				Past				Current <10 cigarettes/day				Current 10+ cigarettes/day			
	Cases/women	RR*	95% g-sCI		Cases/women	RR*	95% g-sCI		Cases/women	RR*	95% g-sCI		Cases/women	RR*	95% g-sCI	
1-2	447/166925	1.00	0.91	1.10	260/77807	1.32	1.17	1.49	46/13653	1.27	0.95	1.70	210/32901	2.62	2.29	3.01
3-6	274/107071	0.97	0.86	1.10	194/63291	1.21	1.05	1.39	53/11045	1.87	1.43	2.44	262/27085	3.96	3.50	4.48
7-14	269/96062	1.07	0.95	1.21	290/82490	1.39	1.23	1.56	78/13390	2.30	1.84	2.87	474/37696	5.24	4.78	5.75
15+	68/20475	1.33	1.05	1.69	129/25774	2.06	1.73	2.45	18/3373	2.21	1.39	3.51	285/12726	9.70	8.63	10.91
p $^{(trend)}$		0.03				<0.001				0.005				<0.001		

* Adjusted for region, body mass index, deprivaton index, educational attainment, strenuous exercise, use of oral contraceptives and menopausal hormones and stratified by year of birth and year completed recruitment questionnaire.

eTable5: Alcohol-BMI interaction analyses restricted to never MHT users

		BMI (kg/m²)				
	Number of cases (<25,25-,≥30)	Relative risk* (95%CI) per 1 drink/day of alcohol <25	Relative risk* (95%CI) per 1 drink/day of alcohol 25-29	Relative risk* (95%CI) per 1 drink/day of alcohol ≥30	LR χ^2 test	$p_{(interaction)}$
Breast (C50)††	8137/6886/3406	1.14 (1.10-1.18)	1.11 (1.07-1.16)	1.13 (1.07-1.19)	0.54	0.764
Cervix (C53)§	175/129/67	0.93 (0.72-1.19)	1.06 (0.80-1.41)	0.90 (0.59-1.39)	0.61	0.737
Endometrium (C54)§	1147/1504/1597	0.97 (0.88-1.07)	1.00 (0.92-1.09)	0.97 (0.88-1.05)	0.40	0.819
Ovary (C56)¶	1348/1088/461	0.88 (0.80-0.96)	1.03 (0.93-1.14)	0.88 (0.74-1.04)	5.65	0.059

* Relative risk and 95% confidence intervals (CI) per 1 drink/day increase in alcohol intake, adjusted by region, deprivation index, educational attainment, smoking, strenuous exercise and use of oral contraceptives and stratified by year of birth and year completed recruitment questionnaire. For estimation of trends, intake categories 1-2, 3-6, 7-14, 15+ drinks/week, were scored according to the mean alcohol intake at reassessment 7.8 years later (2.6, 5.5, 10.0, 17.0 drinks/week respectively).

†† Additional adjustment for age at menarche, age at first birth, parity and family history of breast cancer.

§ Additional adjustment for age at menarche, age at first birth and parity. Includes only women who answered no to hysterectomy at recruitment.

¶ Additional adjustment for age at menarche, age at first birth and parity. Includes only women who answered no to bilateral oophorectomy at recruitment.

eTable6: Alcohol-MHT interaction analyses restricted to women of normal BMI (20-24.9 kg/m²)

		HRT use				
	Number of cases (never/past/current)	Relative risk* (95%CI) per 1 drink/day of alcohol Never	Relative risk* (95%CI) per 1 drink/day of alcohol Past	Relative risk* (95%CI) per 1 drink/day of alcohol Current	LR χ^2 test	$p_{(interaction)}$
Breast (C50)††	7660/2720/8898	1.13 (1.09-1.17)	1.11 (1.04-1.18)	1.11 (1.08-1.15)	0.59	0.744
Cervix (C53)§	164/44/77	0.98 (0.75-1.26)	0.84 (0.51-1.40)	1.19 (0.84-1.68)	1.43	0.488
Endometrium (C54)§	1091/349/876	1.01 (0.92-1.12)	0.90 (0.75-1.07)	0.92 (0.82-1.03)	2.36	0.307
Ovary (C56)¶	1250/402/1121	0.89 (0.81-0.98)	0.99 (0.84-1.17)	1.03 (0.93-1.13)	4.38	0.112

* Relative risk and 95% confidence intervals (CI) per 1 drink/day increase in alcohol intake, adjusted by region, deprivation index, educational attainment, smoking, strenuous exercise and use of oral contraceptives and stratified by year of birth and year completed recruitment questionnaire. For estimation of trends, intake categories 1-2, 3-6, 7-14, 15+ drinks/week, were scored according to the mean alcohol intake at reassessment 7.8 years later (2.6, 5.5, 10.0, 17.0 drinks/week respectively).

†† Additional adjustment for age at menarche, age at first birth, parity and family history of breast cancer.

§ Additional adjustment for age at menarche, age at first birth and parity. Includes only women who answered no to hysterectomy at recruitment.

¶ Additional adjustment for age at menarche, age at first birth and parity. Includes only women who answered no to bilateral oophorectomy at recruitment.
